# Risk factors for secondary Glaucoma in patients with Vogt-Koyanagi-Harada disease

**DOI:** 10.1186/s12348-022-00300-7

**Published:** 2022-07-11

**Authors:** Carlos Alvarez-Guzman, Curt Hartleben-Matkin, Raul E. Ruiz-Lozano, Alejandro Rodriguez-Garcia, Manuel E. Quiroga-Garza, Jorge E. Valdez-Garcia

**Affiliations:** 1 Tecnologico de Monterrey, Escuela de Medicina y Ciencias de la Salud, Instituto de Oftalmologia y Ciencias Visuales, Monterrey, Nuevo Leon Mexico; 2 Fundación de Asistencia Privada Conde de Valenciana, IAP, Ciudad de México, México Chimalpopoca 14. Col. Obrera, CP 06800 Ciudad de México, Mexico; 3 Instituto de Oftalmologia y Ciencias Visuales (1er Piso Ote.) Hospital Zambrano Hellion Tec Salud, Av. Batallon de San Patricio #112. Col. Real de San Agustin. San Pedro Garza Garcia, Nuevo Leon. CP, Mexico, 66278 Mexico

**Keywords:** Angle-closure glaucoma, Immunosuppressive therapy, Peripapillary atrophy, Sunset glow fundus, Vogt-Koyanagi-Harada disease

## Abstract

**Background/purpose:**

Identify the prevalence and risk factors for secondary glaucoma among Mexican-mestizo patients with Vogt-Koyanagi-Harada Disease (VKH).

**Methods:**

Retrospective cohort study analyzing the demographic, clinical, and epidemiological variables. Risk estimates were calculated using a Cox proportional hazards regression model.

**Results:**

One hundred eyes of 50 patients, 44 (88%) women and 6 men (12%) with a median age of 35.5 years (IQR 29–46) and a median follow-up time of 72 months (IQR 13.7–126.7) were analyzed. The prevalence of glaucoma was 20%, with angle-closure accounting for 70% of all cases. Significant clinical risk factors for glaucoma development were a chronic recurrent stage at presentation (RR 2.88, 95% CI 1.11–12.63, *p* = 0.037), ≥ 2 episodes of recurrent anterior uveitis (RR 8.52, 95% CI 2.02–35.92, *p* < 0.001), angle-closure disease (ACD, RR 7.08, 95% CI 2.44–20.48, p < 0.001), iris bombé (RR 5.0, 95% CI 2.10–11.90, p < 0.001), and peripapillary atrophy (RR 3.56, 95% CI 1.43–8.85, p < 0.001). Exposure to > 24 months of oral (RR 9.33, 95% CI 2.21–39.28, p < 0.001) or > 12 months of topical corticosteroids (RR 3.88, 95% CI 1.31–11.46, *p* = 0.007) were associated with an increased likelihood for secondary glaucoma development.

**Conclusion:**

Glaucoma is a frequent complication of VKH, often attributed to mixed pathogenic mechanisms. Chronic disease at presentation, recurrent inflammation, angle-closure mechanisms, iris bombé, and peripapillary atrophy represent clinically significant risk factors for developing secondary glaucoma. Prompt and aggressive steroid-spearing immunosuppressive therapy for adequate inflammation control may lower the risk of glaucoma in VKH.

## Background

Vogt-Koyanagi-Harada disease (VKH) is a primary autoimmune choroiditis characterized by a rapid-onset bilateral granulomatous panuveitis associated with neurologic (headache, meningismus, tinnitus) and integumentary (alopecia, poliosis, vitiligo) manifestations [1, 2]. Choroidal inflammation manifests as serous retinal detachment, optic disc edema, and late depigmentation resulting in a sunset glow fundus (SGF) appearance [3]. The prevalence of VKH in Mexican mestizos is approximately 2.4% [4]. Early combined corticosteroid and immunosuppressive therapy (IMT) can improve visual outcomes by reducing the incidence of associated complications related to longer disease duration and recurrent episodes of inflammation [3]. The most frequent VKH complications are band keratopathy, cataract formation, secondary glaucoma, posterior synechiae, and subretinal fibrosis [5, 6].

The prevalence of secondary glaucoma in VKH disease ranges from 2.6% to 45% [7, 8]. A Mexican VKH case series reported secondary glaucoma developed in 24% of eyes, with most of them (67%) requiring surgical management for intraocular pressure control [9]. Few studies have examined the risk factors associated with secondary glaucoma development in VKH eyes. In an Indian study involving 448 VKH eyes, uveal effusion and an increased recurrence rate were significant risk factors for glaucoma [10]. Other reported risk factors for ocular hypertension (OHT) and glaucoma in a large Chinese population of 1457 patients with VKH included worse visual acuity (VA) at first and last visits, a longer interval between the onset of uveitis and referral, more than three recurrent episodes of inflammation, and posterior synechiae formation [11].

There is convincing evidence that long-term corticosteroid treatment is associated with an increased risk of OHT and secondary glaucoma in patients with uveitis. According to a study of uveitic eyes, a prednisone dose > 7.5 mg/day, periocular steroids in the last three months, > 8 drops/day of topical corticosteroids, and prior use of fluocinolone implants were major risk factors for glaucoma development [12].

This study aims to determine the prevalence of glaucoma associated with VKH disease and identify its clinical and therapeutic risk factors in a cohort of Mexican patients.

## Methods

### Design and setting

A retrospective cohort study on VKH Mexican-mestizo patients was performed at the Ocular Immunology and Uveitis Service between January 2002 and October 2020. The study was approved by our Ethics and Research Committees (License No. P000367-FRGVKH-CEIC CR002), following the tenets of the Declaration of Helsinki.

### Study population

Only patients with a minimum follow-up of 12 months and diagnosed according to the revised diagnostic criteria for VKH from the International Committee on Nomenclature in 2001 were included for analysis [13]. Other forms of uveitis resembling VKH were excluded based on the clinical history, physical and ophthalmologic examinations, and laboratory investigation for infectious diseases.

Clinical data collected included: age, gender, age at diagnosis, extraocular manifestations, symptoms onset, and VKH stage at the time of diagnosis. Patients were classified into two groups based on glaucoma or not at the last visit. Glaucoma was defined as a cup-to-disc ratio larger than 0.7, asymmetry between the two eyes larger than 0.2, or the presence of a nerve fiber layer defect on fundus examination.

### VKH management

At the initial visit, VKH patients in the uveitic or chronic recurrent phase of the disease were treated with oral prednisone (1 mg/kg/day) and topical corticosteroids as a standard management protocol. Simultaneously, IMT was initiated if there were no systemic contraindications or laboratory abnormalities, with azathioprine (2.0–2.5 mg/kg/day), the first-line treatment in all patients. Early IMT was defined as starting therapy within the first 6 weeks of VKH diagnosis at our institution. The total exposure time to oral and topical corticosteroids was calculated for the first 24 months of follow-up time.

### Statistical analysis

IBM Statistical Package for Social Sciences (SPSS) v.21 (IBM Inc., Armonk, NY, USA) was used for statistical analysis. First, normality was assessed with the Kolmogorov-Smirnov test. The demographic characteristics were summarized using means and standard deviations for normally distributed data, whereas medians and interquartile ranges (IQRs) were used for skewed data. Group comparisons were performed using the Mann-Whitney U test, student t-test, or chi-square test. Multivariate logistic regression analysis was performed to identify risk factors for developing secondary glaucoma in non-glaucomatous eyes. The Kaplan-Meier survival curve was plotted at the first visit for glaucoma development in VKH eyes without glaucoma. A *p*-value < 0.05 was considered statistically significant.

## Results

### Sociodemographic characteristics

A total of 50 patients (100 eyes) with VKH disease were divided into two groups: 10 with glaucoma (20 eyes) vs. 40 without glaucoma (80 eyes). The median age was 35.5 years (IQR 29–46), and most patients were women (*n* = 44, 88%). The median follow-up time was 72 months (IQR 13.7–126.7). Headache (76%) and tinnitus (60%) were the most frequent VKH extraocular manifestations reported at the first visit. The baseline characteristics of VKH patients are described in Table 1.

**Table 1:** Baseline characteristics of patients presenting with VKH

**Variable**	**No. patients (%) (*n*** **= 50)**
Female gender	44 (88.0)
Age (years)^a^	35.5 (29–46)
Extraocular findings^b^	
Headache	38 (76.0)
Tinnitus	30 (60.0)
Flu-like symptoms	18 (36.0)
Vitiligo	7 (14.0)
Poliosis	4 (8.0)
Follow-up time (months)^a^	72 (13.7–126.7)
**Variable**	**No. eyes (%) (*n*** **= 100)**
BCVA (LogMAR)^a^	1.0 (0.50–1.52)
IOP (mmHg)	14 (12–16)
Iris nodules	52 (52.0)
VKH stage at presentation	
Uveitic	69 (69.0)
Chronic-recurrent	27 (27.0)
Convalescent	4 (4.0)
Angle configuration	
Open	69 (69.0)
Closed	31 (31.0)
VKH signs (*n* = 98)^bc^	
Sunset glow fundus	26 (26.5)
Dalen-Fuchs nodules	37 (37.8)
Serous retinal detachment	47 (48.0)
Peripapillary atrophy	20 (20.4)
Optic disc swelling	48 (49.0)
C/D ratio (*n* = 35)^ad^	0.4 (0.3–0.5)

*VKH* Vogt-Koyanagi-Harada, *IQR* interquartile range, *BCVA* best-corrected visual acuity, *LogMAR* logarithm of the minimum angle of resolution, *C/D* cup-to-disc ratio

^a^Median (IQR)

^b^Several patients and/or eyes had > 1 features

^c^Dense cataract precluded fundus evaluation (*n* = 2)

^d^Presence of papillitis, peripapillary atrophy, or opaque media precluded fundus evaluation (*n* = 65)

### Ocular findings at baseline

At the first visit, the median best-corrected visual acuity (BCVA) was 1.0 (IQR 0.50–1.52) LogMAR. Most eyes (69%) presented during the uveitic stage, followed by the chronic-recurrent (27%) stage. Sixty-nine (69%) eyes had an opened iridocorneal angle, whereas 31% of eyes had a closed angle, defined as 180 degrees or more of iridotrabecular contact without indentation on gonioscopy [14]. Most eyes had one or more typical fundoscopic signs of VKH disease. Optic disc swelling (49%), serous retinal detachment (48%), and Dalen-Fuchs nodules (38%) were the most observed. A dense cataract precluded fundus evaluation in only two eyes (Table 1).

### Outcome comparative analysis between glaucoma versus non-glaucoma VKH eyes

There was a difference in the extraocular findings between groups. Six eyes with glaucoma (30%) developed vitiligo compared to only 8 (10%) eyes without glaucoma (*p* = 0.021). While 13 eyes (65%) of the glaucoma group presented in the chronic recurrent phase of VKH disease, only 14 eyes (17.5%) did from the non-glaucoma group (*p* < 0.001).. In glaucoma eyes, the time required to achieve inflammatory control (*p* = 0.026) and the time elapsed to the first VKH disease recurrence (p < 0.001) was significantly longer (Table 2). The total time of topical corticosteroid exposure (62 vs. 11.5 months, *p* = 0.008) and oral prednisone (25 vs. 8.5 months, p < 0.001) was significantly longer in the glaucoma group. At the last visit, classic VKH signs, including sunset glow fundus (*p* = 0.031), Dalen-Fuchs nodules (*p* = 0.001), peripapillary atrophy (PPA, *p* = 0.002), and papillitis (p < 0.001) were more prevalent in glaucoma eyes.

**Table 2:** Comparative analysis of VKH patients baseline demographic data and clinical features of VKH eyes with and without glaucoma

Baseline characteristics	Non-glaucoma (***n*** = 80 eyes, %)	Glaucoma (***n*** = 20 eyes, %)	***p*** Value^**d**^
Female gender	68 (85.0)	20 (100.0)	0.327
Age (years)^a^	35 (26.7–45.7)	37 (29–54)	0.304
**Extraocular findings**^**b**^			
Poliosis	2 (5.0)	2 (20.0)	0.049
Vitiligo	4 (10.0)	3 (30.0)	0.021
Flu-like	14 (35.0)	4 (40.0)	0.600
Headache	30 (75.0)	8 (80.0)	0.640
Tinnitus	25 (62.5)	5 (50.0)	0.307
**VKH stage at presentation**			
Uveitic	62 (77.5)	7 (35.0)	< 0.001
Chronic-recurrent	14 (17.5)	13 (65.0)	< 0.001
Convalescent	4 (5.0)	0 (0)	< 0.001
Angle configuration			
Open	64 (80.0)	5 (25.0)	< 0.001
Closed	16 (20.0)	15 (75.0)	< 0.001
**Outcomes at last visit**			
BCVA (LogMAR)^a^	0.2 (0–0.6)	0.55 (0.22–2.3)	0.003
**VKH signs (*n*** **= 98)**^**b**^			
Sunset glow fundus	50 (62.5)	16 (88.9)^c^	0.031
Dalen-Fuchs nodules	58 (60)	18 (100)^c^	0.001
Serous retinal detachment	2 (2.5)	1 (5.6)^c^	0.460
Peripapillary atrophy	39 (37.5)	14 (77.8)^c^	0.002
Papillitis	2 (2.5)	0 (0)	< 0.001
C/D ratio (*n* = 35)^ad^	0.4 (0.3–0.4)	0.8 (0.55–0.9)	< 0.001
Recurrence (yes)	44 (55.0)	15 (75.0)	0.130
Time to achieve inflammatory control (months)^a^	2 (1–3)	3 (2–3)	0.026
Time to first recurrence (months)^a^	5 (2–8)	18 (8–48)	< 0.001
Exposure topical steroids (months)^a^	8.5 (2.2–24)	25 (14–54)	0.008
Exposure oral prednisone (months)^a^	11.5 (7–26.7)	62 (39–104)	< 0.001

*VKH* Vogt-Koyanagi-Harada, *IQR* interquartile range, *BCVA* best-corrected visual acuity, *LogMAR* logarithm of the minimum angle of resolution, *C/D* cup-to-disc ratio

^a^Median (IQR)

^b^Several patients and/or eyes had > 1 features

^c^Dense cataract precluded fundus evaluation in 2 eyes from the glaucoma group

^d^The presence of papillitis, peripapillary atrophy, or opaque media precluded fundus evaluation

^d^*p*-value > 0.05 is considered statistically significant

**Fig. 1:**
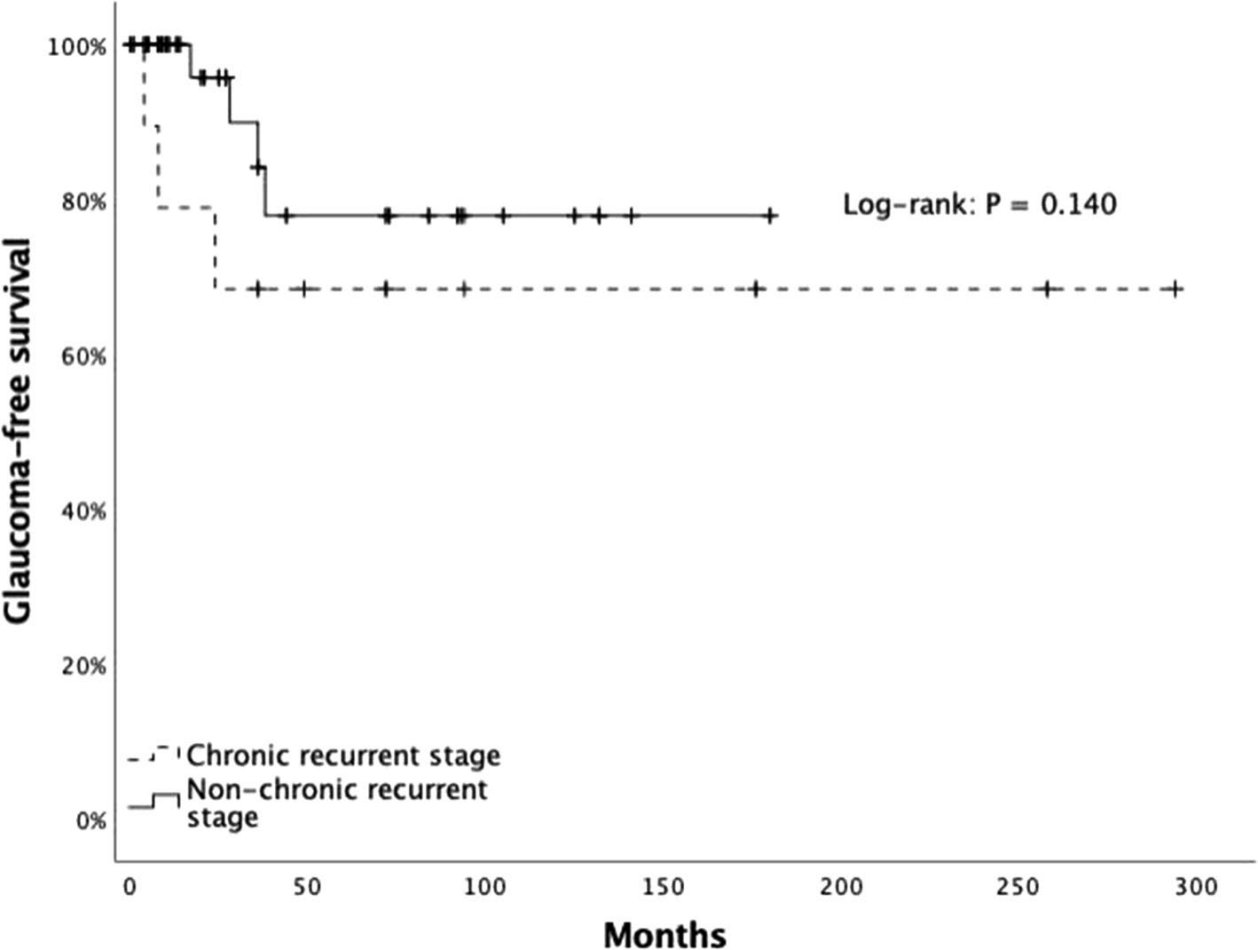
Kaplan-Meier plot of secondary glaucoma development based on the VKH phase at first visit. The incidence of glaucoma in patients with chronic-recurrent VKH disease at one and two years were 22.1% and 31.6%, respectively. Patients with uveitis VKH disease had a risk of secondary glaucoma development of 22.1%. This difference was not significant (*p* = 0.140)

Kaplan-Meier survival analysis was done for the development of glaucoma based on the VKH phase at presentation. Figure 1 shows the cumulative probabilities of developing glaucoma. None of the eyes within the acute uveitic or convalescence stage at presentation developed glaucoma in the first and second years, whereas only 22.1% developed glaucoma in the fifth year. On the other hand, 21.1% of eyes in the chronic recurrent phase of the disease at the initial visit developed glaucoma in the first year and 31.6% in the second year. Multivariate logistic regression analysis to determine the effect of oral and topical corticosteroids, early IMT, the stage of VKH disease at diagnosis, and clinical characteristics on the likelihood of developing secondary glaucoma are depicted in Table 3. Exposure to topical corticosteroids for more than 12 months (RR 3.88, 95% CI 1.31–11.46, *p* = 0.007) or oral prednisone for more than 24 months (RR 9.33, 95% CI 2.21–39.28, *p* < 0.001) were major treatment risk factors for glaucoma. Reduced risk for glaucoma was not found when early IMT was started within 6 weeks of the symptom onset in our population (RR 0.40, 95% CI 0.12–1.30, *p* = 0.183).

**Table 3:** Multivariate analysis of risk factors for secondary glaucoma in eyes with VKH disease

Risk Factor	Non-glaucoma (***n*** = 78)^a^	Glaucoma (***n*** = 16)^b^	Relative Risk (95% CI)	***p*** Value^**c**^
Oral prednisone > 24 months	24 (30.8)	12 (85.7)	9.33 (2.21–39.28)	< 0.001
Topical steroids > 12 months	26 (33.3)	10 (71.4)	3.88 (1.31–11.46)	0.007
Anterior chamber cells ≥2+	37 (47.4)	11 (78.6)	3.36 (1.003–11.26)	0.032
≥2 recurrences	26 (33.3)	12 (85.7)	8.52 (2.02–35.92)	< 0.001
Chronic-recurrent stage	13 (16.7)	6 (42.9)	2.88 (1.11–12.63)	0.037
Angle-closure	14 (18.9)	10 (71.4)	7.08 (2.44–20.48)	< 0.001
Iris bombé	6 (7.7)	6 (42.9)	5.00 (2.10–11.90)	0.002
Peripapillary atrophy	10 (12.8)	6 (42.9)	3.56 (1.43–8.85)	0.014

*VKH* Vogt-Koyanagi-Harada

^a^Two eyes without management with oral prednisone (convalescent stage) were excluded

^b^Four eyes with glaucoma at first visit were excluded

^c^*p*-value > 0.05 is considered statistically significant

## Discussion

Secondary glaucoma is one of the most feared complications of VKH disease. Consistent with other reports, the present study findings suggest that the chronic recurrent phase of the VKH disease [15, 16], significant anterior chamber reaction (> 2+ cells) [16, 17], multiple inflammatory recurrences, angle-closure disease (ACD) [18, 19], PPA, the presence of extraocular manifestations, and a worse VA at disease onset [17], are deemed as significant risk factors for secondary glaucoma development. Furthermore, patients who develop OHT or glaucoma have the worst visual prognosis [10, 11].

Open-angle mechanisms associated with corticosteroid-induced OHT and inflammatory trabecular meshwork dysfunction are considered the leading causes of glaucoma in VKH [19]. However, the role of angle-closure mechanisms has gained significant interest nowadays [20]. Clinical signs of ACD are the development of peripheral anterior synechiae, seclusio pupillae, iris bombé configuration, and ciliary body detachment with anterior rotation of ciliary processes [11]. Many studies report that the chronic recurrent stage of the disease and multiple disease recurrences, both increased in our glaucoma group, are associated with angle-closure glaucoma [19]. Yang et al. reported a 51% prevalence of ACD among VKH patients who developed OHT/glaucoma [11]. The authors report that acute angle-closure glaucoma at onset (11%), peripheral anterior synechiae (11%), and seclusio pupillae (29%) were the mechanisms of angle-closure [11]. We found a higher prevalence (71%) of ACD among glaucoma eyes. We believe this finding might be associated with our more permissive definition of angle closure. Aside from initial aggressive immunotherapy, routine gonioscopy must be performed in VKH patients at every visit to look for ACD, preventing glaucoma development.

The optimal management of acute VKH with a combination of systemic corticosteroids and a nonsteroidal immunosuppressive agent is critical for avoiding disease progression to the chronic recurrent phase, which may occur in up to two-thirds of patients [21]. Disease progression, recurrence of inflammation, and complication development may be avoided if prompt and adequate treatment is started within three weeks of symptoms onset [3]. The Kaplan-Meier analysis shows a clear difference between VKH phases, where none of the eyes seen during the uveitic phase of the disease developed glaucoma in the first and second year of follow-up, highlighting the importance of prompt and aggressive treatment in this phase of VKH. Furthermore, only 22.1% of eyes presenting in the uveitic stage developed glaucoma after a 5-year follow-up. In contrast, the same incidence of glaucoma was found in eyes seen at the chronic recurrent stage but in the first year of follow-up. These findings are supported by Al-Kharashi et al., who reported that rapid corticosteroid tapering, defined as 1 mg/kg of oral prednisone for less than 2 months or tapering to 10 mg in less than two months, was associated with > 3 recurrences. Moreover, the authors reported that multiple recurrences were significantly associated with a higher incidence of glaucoma development [17].

Evidence suggests that increased exposure to systemic corticosteroids is associated with PPA [22]. Moreover, the development of PPA is significantly associated with an increased risk of either cataract, glaucoma, or subretinal neovascular membrane formation [16]. In our study, PPA has been deemed a significant risk factor for glaucoma development, and prolonged topical (> 12 months) and systemic (> 24 months) corticosteroid exposure were also significantly associated with developing glaucoma. Thus, recognizing that oral prednisone exposure might represent a modifiable risk factor for glaucoma emphasizes the critical role of early IMT.

SGF is the loss of choroidal melanocytes after inflammatory T-cell infiltration that develops during the convalescent stage of VKH disease, typically 2 to 6 months after onset [5, 7, 23]. It is considered a marker of ongoing choroidal inflammation [24]. El-Asrar et al. reported that early IMT significantly reduces the risk of SGF and that SGF is associated with the development of ocular complications, including glaucoma, cataract, and subretinal neovascular membrane formation [16]. However, when analyzing glaucoma alone, these authors found no significant differences in the occurrence of secondary glaucoma in acute (*p* = 0.227) and/or chronic-recurrent (*p* = 0.241) VKH patients receiving IMT [16]. We found a significantly higher prevalence of SGF in eyes developing glaucoma at the initial (*p* = 0.002) and final (*p* = 0.031) visits. However, SGF failed to become a risk factor for secondary glaucoma development (RR 2.53, 95% CI 0.99–6.49, *p* = 0.08). Moreover, we could not demonstrate that early IMT per se reduced the risk for developing glaucoma. Interestingly, Urzua et al. could not find significant differences in VA improvement or reduced risk of ocular complications (i.e., glaucoma, OHT, cataract) in patients with or without early IMT (< 6 weeks) [25].

A probable explanation of why early IMT did not confer a reduced risk for the development of secondary glaucoma in our series is that one-third of the eyes within our cohort came in the chronic-recurrent stage of VKH disease. Reinforcing this assumption, of the 16 eyes that developed glaucoma during the follow-up time, 43% were in the chronic-recurrent phase of the disease.. Moreover, our results are consistent with other studies reporting an increased risk of secondary glaucoma in patients with chronic-recurrent VKH [15, 16].

Finally, the retrospective nature of the study design, the limited number of patients included, and the fact that not all clinical and therapeutic risk factors for glaucoma were considered for analysis precluded us from drawing definitive conclusions about this finding. Also, the selection bias of Hispanic patients with a usually larger proportion of severe and advanced diseases referred to tertiary referral centers may not represent the general population of VKH disease in Mexico. Nevertheless, our findings are valuable, showing relation to ethnic and environmental factors that can further influence the response to anti-inflammatory treatment and the development of glaucoma.

In conclusion, these findings have important implications for the clinical management of VKH patients. Prompt and combined systemic corticosteroid with IMT for active disease, detecting early manifestations of angle-closure, and limiting topical and oral corticosteroid exposure are crucial to reducing secondary glaucoma development in patients with VKH disease. The recognition of glaucoma as a prominent complication of chronic progressive disease could eventually encourage adequate inflammatory control to prevent a poor visual outcome.

## Data Availability

Results obtained in this study were generated from data collected and analyzed based on the stated methods section. Since all data is already found in the manuscript, there are no supplementary files.

## Abbreviations

ACD: angle-closure disease
BCVA: best-corrected visual acuity
CI: confidence interval
IMT: immunosuppressive therapy
IQR: interquartile range
LogMAR: logarithm of the minimum angle of resolution
MMF: mycophenolate mofetil
OHT: ocular hypertension
PPA: peripapillary atrophy
RR: relative risk
SGF: sunset glow fundus
SPSS: Statistical Package for Social Sciences
VA: visual acuity
VKH: Vogt-Koyanagi-Harada
